# Supplementary figures and images for: Molecular structures and conformations of protocadherin-15 and its complexes on stereocilia elucidated by cryo-electron tomography
Source: eLife. 2021 Dec 29;10:e74512. doi: 10.7554/eLife.74512 (PMC8776254; doi:10.7554/eLife.74512)

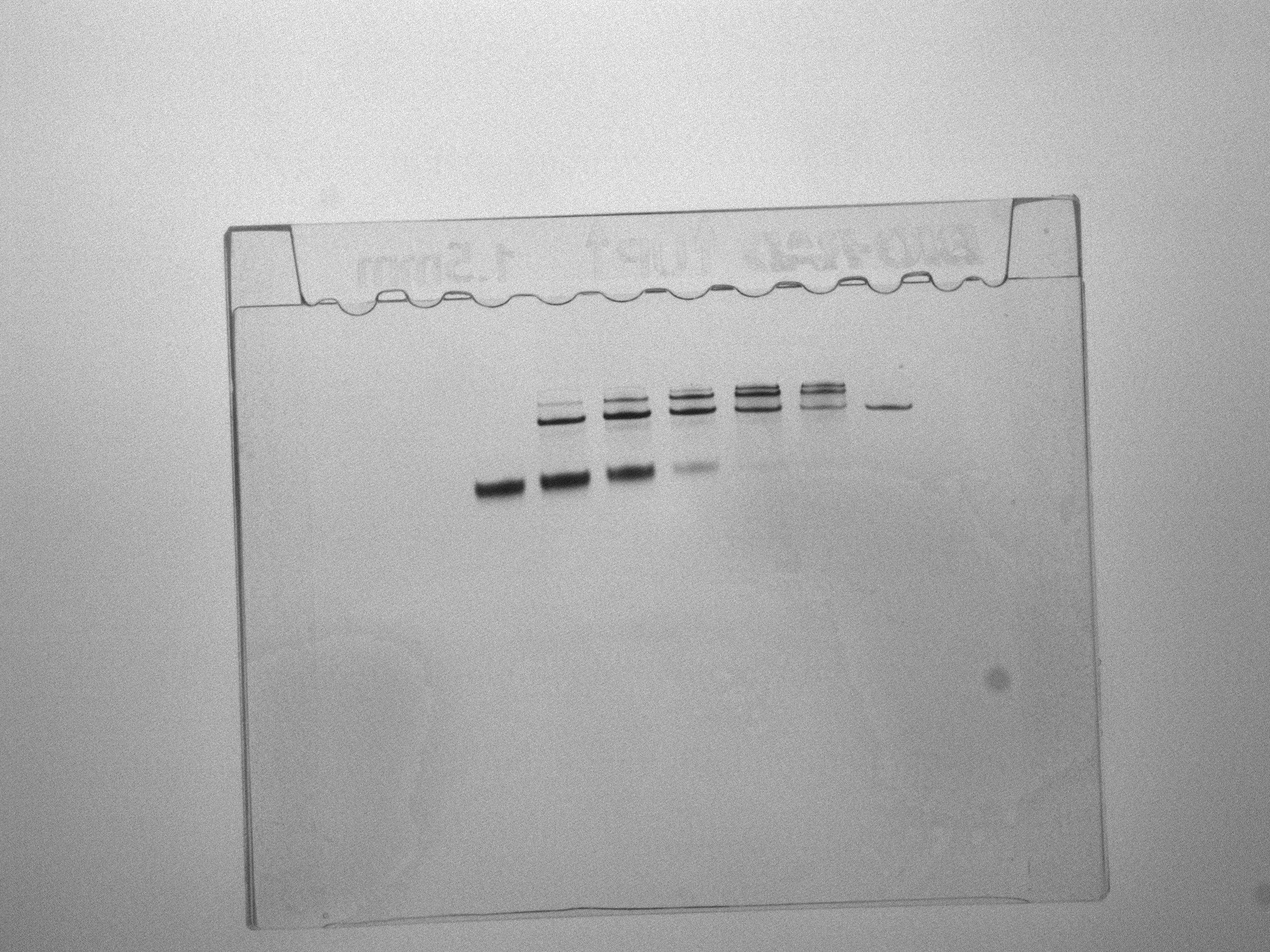

Supplement: Figure 2—source data 1. [file elife-74512-fig2-data1.zip › Figure 2-source data.TIF]
